# Development of cardiac risk prediction model in patients with HER-2 positive breast cancer on trastuzumab therapy

**DOI:** 10.1186/s40959-023-00177-y

**Published:** 2023-05-19

**Authors:** Prince Otchere, Olusola Adekoya, Samuel B. Governor, Naveen Vuppuluri, Akruti Prabhakar, Stella Pak, Oduro Oppong-Nkrumah, Francis Cook, Rudy Bohinc, Gregory Aune

**Affiliations:** 1 The University of Texas Health Science Center at San Antonio, San Antonio, Texas, USA; 2 Kettering Medical Center, Kettering, OH USA; 3 Washington University School of Medicine, St. Louis, MO USA; 4 Wright State University, Dayton, OH USA; 5 Albany Medical Center, Albany, NY USA; 6 McGill University, Montreal, QC Canada; 7 Harvard University, Cambridge, MA USA

**Keywords:** Predictive Model, Cardiotoxicity, Trastuzumab, Breast Cancer

## Abstract

**Background:**

25% of all breast cancer patients have HER-2 overexpression. Breast Cancer patients with HER-2 overexpression are typically treated with HER-2 inhibitors such as Trastuzumab. Trastuzumab is known to cause a decrease in left ventricular ejection fraction. The aim of this study is to create a cardiac risk prediction tool among women with Her-2 positive breast cancer to predict cardiotoxicity.

**Method:**

Using a split sample design, we created a risk prediction tool using patient level data from electronic medical records. The study included women 18 years of age and older diagnosed with HER-2 positive breast cancer who received Trastuzumab. Outcome measure was defined as a drop in LVEF by more than 10% to less than 53% at any time in the 1-year study period. Logistic regression was used to test predictors.

**Results:**

The cumulative incidence of cardiac dysfunction in our study was 9.4%. The sensitivity and specificity of the model are 46% and 84%, respectively. Given a cumulative incidence of cardiotoxicity of 9%, the negative predictive value of the test was 94%. This suggests that in a low-risk population, the interval of screening for cardiotoxicity may be performed less frequently.

**Conclusion:**

Cardiac risk prediction tool can be used to identify Her-2 positive breast cancer patients at risk of developing cardiac dysfunction. Also, test characteristics in addition to disease prevalence may inform a rational strategy in performing cardiac ultrasound in Her-2 breast cancer patients. We have developed a cardiac risk prediction model with high NPV in a low-risk population which has an appealing cost-effectiveness profile.

## Introduction

An estimated 25% of all breast cancer patients have HER-2 overexpression [1]. Breast Cancer patients with HER-2 overexpression are typically treated with HER-2 inhibitors such as Trastuzumab. Other HER-2 inhibitors in clinical use include lapatinib, trastuzumab emtansine (T-DMI) and pertuzumab [2].

Trastuzumab is known to cause a decrease in left ventricular ejection fraction (LVEF) in about 3%-19% of patients undergoing treatment [3, 4]. Symptomatic heart failure occurs less frequently than asymptomatic LVEF decline [5].

One approach to prevent cardiotoxicity is predicting patients who are at risk of cardiac toxicity to mitigate against such risk. Currently, there is no widely accepted cardiac risk prediction tool in clinical practice in HER-2 positive breast cancer patients undergoing therapy with trastuzumab. A previous study developed a risk prediction model to predict the risk of cardiotoxicity among older women between 67 and 94 years old. The outcome of interest for their study was development of heart failure or cardiomyopathy within 3 years after diagnosis [6]. Also, the study used administrative data from the SEER-Medicare Database. The study outcome, heart failure, was determined by a billing code which lacked granular information about outcome definition [6, 7].

The aim of this study is to create a cardiac risk prediction tool among women with HER-2 positive breast cancer to identify the individuals unlikely to develop Cancer therapy-related cardiac dysfunction (CTRCD). In our study, CTRCD was defined as a drop in LVEF by more than 10% to less than 53% at any time in the first year following trastuzumab therapy.

## Methods

### Study design

We conducted a retrospective cohort study using electronic medical records from the Kettering Health Network, Ohio, which consisted of 358 participants, and Premier Health Network, Ohio, which included 119 participants. The use of Trastuzumab was identified by reviewing the electronic medical record. We combined the two datasets into a total sample size of 477 participants.

### Study participants

The study included women 18 years of age and older diagnosed with HER-2 positive breast cancer who received Trastuzumab between 2015 and 2019. Patients with echocardiograms with LVEF obtained at the start of chemotherapy and the end of chemotherapy were included in the study. Patients with no baseline echocardiogram or who had a cardiac dysfunction at baseline, defined as an LVEF less than 53%, were excluded from the study [7]. If a participant did not have an echocardiogram at 12 months after initiating therapy, but had an echocardiogram at 3 months, 6 months, or 9 months from the baseline echocardiogram, the patient was retained, and the last echocardiogram was adopted as the final echocardiogram. The final sample size used in our analysis was 415 after we excluded participants who did not meet our inclusion criteria.

### Outcome measure

Outcome measure was defined as a drop in LVEF by more than 10% to less than 53% at any time in the first year following trastuzumab therapy [7].

### Construction of predictors

In our analysis, candidate predictors of cardiotoxicity known from previous studies including age, race, history of coronary artery disease, history of prior radiation use, concurrent use of anthracycline, current radiation therapy, diabetes mellitus, hypertension and renal disease were tested [4, 6].

### Statistical analysis

The sample was split into a derivation cohort consisting of 65% of the study population and 35% of the sample was used as a validation cohort. Logistic regression was used to test predictors mentioned earlier. Receiver operating curve was used to determine the area under the curve, and confusion matrix analytics was used to determine the sensitivity, specificity, accuracy of predictions, negative predictive value, and positive predictive value of cardiac dysfunction in our study. We used 0.09 or 9% as the cutoff point for defining the predicted probability as an event. Data analysis was performed in R statistical software (version 4.2).

### IRB

The study was approved by the IRB of individual institutions. The study was considered exempt and thus informed consent was not required.

## Results

The baseline characteristics of the study population are outlined in Table 1. A total of 415 patients were included in the analysis. The average age of the study population was 58.4 years. Mean age did not differ by cardiotoxicity. Baseline left ventricular ejection fraction was 62.6%. The cumulative incidence of cardiac dysfunction in our study was 9.4%. The prevalence of diabetes mellitus was 14.9% of the study population. 40% of the study population was exposed to radiation. 25 patients were exposed to Adriamycin, and this accounted for 6.1% of the study population. Among participants who developed cardiac dysfunction, 28.9% of them had diabetes, whereas, among those who did not have cardiac dysfunction, only 13.9% of them had diabetes. Diabetes showed a statistically significant difference between those who developed cardiac dysfunction versus those who did not. Adriamycin therapy was also statistically significant between those who developed cardiac dysfunction versus those who did not. Among those who developed cardiac dysfunction, 15.4% had received Adriamycin whereas of those who did not develop cardiac dysfunction, only 5.1% had received Adriamycin therapy. Hypertension was prevalent in 48% of the study population. Coronary artery disease had a prevalence of 8%.

**Table 1:** Baseline characteristics of patients included in the cardiac risk prediction model

**Descriptive Statistics of the full data** Descriptive Statistics of Training Data Set in our Sensitivity Analysis				
		Incident cardiac dysfunction		
	Overall	No	Yes	*P*-value
n	415	376	39	
Age.at.Dx (mean (SD))	58.40 (12.85)	58 43 (13.00)	58.13 (11.48)	0.891
Baseline.EF (mean (SD))	62.64 (5.75)	62.65 (5.83)	62.52 (4.92)	0.893
Stroke = 1 (%)	17 (4.1)	14 (3.8)	3 (7.9)	0.430
Diabetes = 1 (%)	61 (14.9)	50 (13.4)	11 (28.9)	0.020
Race.1 = white (%)	363 (87.9)	327 (87.4)	36 (92.3)	0.529
Radiation = 1 (%)	166 (40.0)	151 (40.2)	15 (38.5)	0.973
Adriamycin = 1 (%)	25 (6.1)	19 (5.1)	6 (15.4)	0.029
HTN = 1 (%)	200 (48.9)	178 (48.0)	22 (57.9)	0.320
CHF = 1 (%)	10 (2.4)	9 (2.4)	1 (2.6)	1.000
CAD = 1 (%)	33 (8.0)	28 (7.5)	5 (13.2)	0.367
Afib = 1 (%)	20 (4.9)	20 (5.4)	0 (0.0)	0.285
Renal.failure = 1 (%)	23 (5.6)	19 (5.1)	4 (10.5)	0.311
Tobacco.Hx (%)				0.837
Current smoker	58 (14.0)	53 (14.1)	5 (12.8)	
Former smoker	101 (24.3)	90 (23.9)	11 (28.2)	
Never smoked	256 (61.7)	233 (62.0)	23 (59.0)	

### Model derivation

In our analysis, 65% of our data was randomly sampled as a derivation dataset consisting of 270 participants, and 35% as a validation dataset consisting of 145 participants. In our derivation cohort, incident cardiac dysfunction cases consisted of 25 cases (9.3%), and the controls were 245 (90.7%). Descriptive statistics of training data set in our primary analysis is shown in Table 2.

**Table 2:** Descriptive statistics of training data set in our primary analysis

		Incident Cardiac Dysfunction		
	Overall	No	Yes	*P*-value
n	270	245	25	
Age at Dx (mean (SD))	57.69 (12.95)	57.80 (13.11)	56.56 (11.39)	0.649
Stroke (%)	13 (4.9)	11 (4.6)	2(8.0)	0.786
Diabetes (%)	37 (13.9)	30 (12.4)	7(28.0)	0.066
Adriamycin (%)	16 (6.0)	11 (4.6)	5(20.0)	0.008

**Fig. 1:**
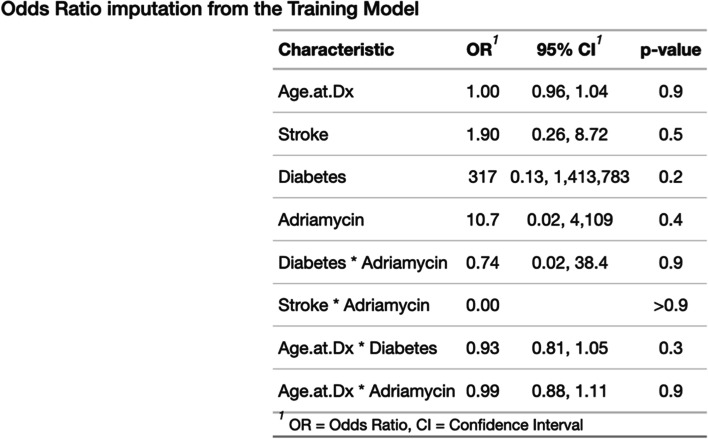
Derivation Model. Shows the derivation model with cardiac risk predictors (possible confounding factors)

Figure 1 shows the derivation model. In the model, the predictors that increased cardiac dysfunction risk included stroke, diabetes, and Adriamycin. Interaction term was used account for effect modification in several predictors.

**Fig. 2:**
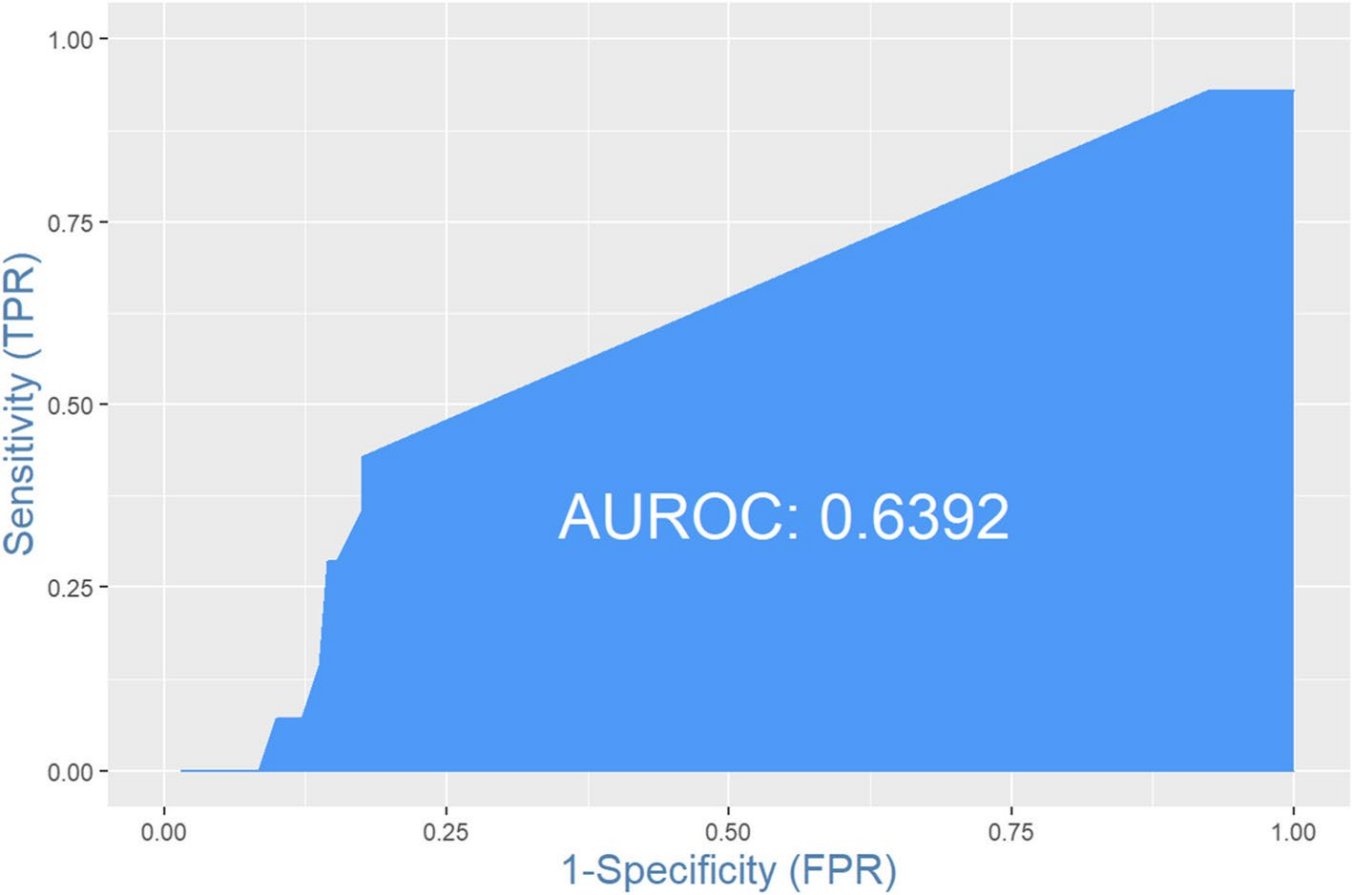
Receiving Operating Curve of the Prediction Model (ROC). The area under the curve (AUC) from Fig. 2. was 64%, indicating that the predictive model outperformed a random guess for determining which patient is likely to develop cardiac dysfunction within a year of trastuzumab therapy

The area under the curve (AUC) from Fig. 2. was 64%, indicating that the predictive model outperformed a random guess for determining which patient is likely to develop cardiac dysfunction within a year of trastuzumab therapy.

We validated our derivation model against our validation dataset. The accurate prediction of our model was 80% with 95% CI (73%—86%), and the sensitivity was 46% and specificity 84%. The positive predictive value of cardiac dysfunction in our study was 22%, and the negative predictive value was 94%. Table 2 shows the performance of our validated derivation model.

## Discussion

In our study, the cumulative incidence of cardiotoxicity was 9.4%. The predictors in the model that increased cardiac dysfunction risk included stroke, diabetes, and Adriamycin. The area under the curve (AUC) in our predictive model was 64%. When the derivation model was validated against our validation dataset, the sensitivity was 46% and the specificity was 84%. However, given a cumulative incidence of 9%, the negative predictive value of the test was 94%. Thus, our model indicates that the probability of not experiencing cardiotoxicity in 12 months given that a patient has a negative echocardiogram result is 94% as shown in Table 3.

**Table 3:** Confusion matrix and statistics of the prediction model

Sensitivity	46%
Specificity	84%
Positive Predictive Value (PPV)	22%
Negative Predictive Value (NPV)	94%
Prevalence	9%

The cumulative incidence of cardiotoxicity in our study was 9.4%, which was higher than the cumulative incidence found in an earlier risk prediction tool derived from the National Surgical adjuvant Breast and Bowel Project which had a cumulative incidence around 4% [8]. The 3-year heart failure/cardiomyopathy rate in another risk prediction study that used SEER-Medicare database in older women with average age of 73 was 19% [6]. The wide range of cardiotoxicity reported in previous studies, 4% and 19% may reflect the differences in outcome assessment in administrative database that relies on billing codes that may not always reflect patient status. Also, the higher cumulative incidence of Heart failure/Cardiomyopathy in the risk prediction tool that used SEER-Medicare database may also be due to the increased average age of the study population 73 years [6].

The current risk prediction tool shows promise with a high specificity and moderate sensitivity for predicting cardiotoxicity in HER2 patients undergoing cancer therapy. Given the cumulative incidence of 9% of cardiotoxicity among HER2 women undergoing cancer therapy in a one-year follow-up, this means that about 91% of the women do not develop cardiotoxicity within a year of follow-up. With our model, we hope to be able to identify women who are at high risk of developing cardiotoxicity from those who are not at increased risk for cardiotoxicity. This way, high-risk women for cardiotoxicity can be screened more frequently and perhaps according to the current practice of imaging patients every 3 months [9], whereas the low-risk women can be screened less frequently. This approach has a more appealing cost-effectiveness profile while being safe as against the current practice of screening every patient rigorously, whereas just a few only develop cardiotoxicity within a year of follow-up. This is an important finding that may suggest that in low-risk populations, the interval of screening for cardiotoxicity may be performed once a year safely.

Concurrent use of trastuzumab and anthracycline is rare due to the concern for their combined effect on cardiotoxicity at 27% [10]. For this reason, sequential use of anthracycline after trastuzumab is the standard of care [11]. Thus, we included “concurrent use of trastuzumab and anthracycline) as a variable in the construction of our model.

One of the main limitations of our study was the small sample size. We hope to in the future expand our sample size so that we can optimize the performance of our model by increasing its sensitivity and specificity. The small sample size also resulted in the wide confidence interval for the diabetes variable. Repeating this study with a larger sample would allow us to get a more precise confidence interval.

Also, not all participants were followed for the entire study duration of a 12-month. Some of the participants were lost to follow-up and therefore did not have an LVEF at some of the time points. Because of the different follow-up times for participants, the loss of follow-up could introduce attrition bias into our study.

We could not differentiate the patients who underwent whole breast radiation from those who received partial breast radiation due to frequent missing data in the electronic medical record. We were also unable to separate the group with metastatic disease and those with curative intent in our study cohort. Repeating the study with prospective data collection would allow us to include the variables in the analysis.

In our study with 415 patients, 386 patients underwent the surveillance echocardiogram every 3 months through 12 months. We had only 29 individuals who received the echocardiographic screening less frequent than 3 months intervals. Follow-up studies with a larger group of those who had less frequent surveillance interval than 3 months would improve both internal and external validity of the study outcome. Inclusion of the patients treated with Emtasine, Pertuzumab, and other Trastuzumab-related medications would help increase the sample size.

In addition, GLS data was inconsistently collected at the beginning of the study. As a result, GLS was not included as an endpoint. More intensive supervision of data collection process for follow-up study would allow the collection of the GLS data.

The study findings suggest that imaging yearly may be a reasonable approach to assess cardiotoxicity in a safe manner. For this approach to be adopted widely, this study should be replicated to verify findings. Also, more studies will need to be conducted to determine prevalence levels of toxicity in different geographic locations to better inform local decision.

Since 2019, Dr. Yu and his colleagues have been prospectively collecting data to develop a risk-based approach to cardiotoxicity surveillance during HER2-positive breast cancer treatment [12]. The dataset from our study can potentially be used to validate the study outcome of the study.

## Conclusion

Cardiac risk prediction tool can be used to identify HER-2 positive breast cancer patients at risk of developing cardiac dysfunction. The test characteristics in addition to disease prevalence may inform our choice on rational strategy in performing cardiac ultrasound in HER-2 positive breast cancer patients. We have developed a cardiac risk prediction model with high NPV in a low-risk population. Future studies should continue to develop risk models with well-defined outcomes using advanced methodologies such as machine learning. Also, further studies to establish imaging interval based in sound research are needed.

## Data Availability

All data and materials are available for review upon request in the shared department drive.
